# Traumatic Injuries of the Brain and Its Membranes

**Published:** 1898-03

**Authors:** 


### Book Reviews.

Traumatic Injuries of the Brain and Its Membranes. With a special study of pistol-shot wounds of the head in their medico-legal and surgical relations. By Charles Phelps, M. D., Surgeon to Bellevue and St. Vincent's Hospitals. Octavo, pp. xiv.—582. With forty-nine illustrations. New York: D. Appleton & Co. 1897.

In these days of books written by authors possessing insufficient experience to qualify them for the work, it is refreshing to read such a masterly treatise as that given us by Dr. Phelps, based as it is upon an experience of 500 cases.

The work is divided into two parts, the first treating of traumatic injuries of the brain and its membranes; the second, of pistol-shot wounds of the head in their medico-legal and surgical relations. Each part rivals the other in the quality and perfection of its contents. In part one the author begins with a preliminary consideration of cranial fractures, based upon a study of 180 cases, in which, as elsewhere in the book, originality is conspicuous. After studying the mechanism of fractures, particularly the basal forms, the author comes to the conclusion that the mechanism is not always the same and that some of them formerly attributed to *contre-coup* are of variable as well as questionable origin; that whether produced by insupportable distortion of the elastic vault, or by transmission of force through osseous structure to the point of discharge of same, or propagated by undulations in the brain substance is not yet known.

Fractures are not significant as osseous lesions, but because of their liability to intracranial complications. As the author tersely puts it, it is the complication and not the fracture which dominates the case. These complications include lacerations, contusion, hemorrhage and septic inflammation in both brain and meninges, to all of which is added hemorrhage from osteal vessels. The symptomatology and diagnosis are then considered. By tracing the fissure from its origin in the vault, by noting the situation of external hemorrhages, serous discharges and extrusions of brain matter, by observing the location of pain and the concurrence of complicating intracranial lesions, the author believes that most cases of basal fracture may be accurately diagnosticated.

From the prognostic point of view cranial fractures in themselves are only exceptionally of importance. The author says their prognosis is really the prognosis of their complications. The fatalities are usually due to concomitant lesions. The treatment of the fracture is local; of the complication, general. Shock contra-indicates local interference (control of hemorrhage excepted). In the words of the author, the general principle that the resort to local measures must await the restoration of nervous and vascular force, except for the relief of hemorrhage by which depression is prolonged, is a fundamental law in surgery. After reaction is established, then primary rather than secondary operation is indicated and its performance becomes imperative. In case of doubt, direct inspection and touch of the injured parts are advised, depressed portions raised, clots removed and the like.

The chapter upon pathology is most clear and satisfying. The intracranial and traumatic lesions are classified primarily as hemorrhages, thromboses of sinuses, contusions, lacerations and their sequelæ, as: meningeal and parenchymatous inflammations (usually septic) and atrophy. What the author has to say under these headings is so condensed and important that direct quotation rather than an abstract is necessary to its proper appreciation. Such quotation space forbids and the reader must be referred to the work itself.

Two chapters are devoted to symptomatology, in which attention is first of all directed to the fact that symptoms without exception result from demonstrable organic change. They are grouped under the name of their pathogenic lesion and include (1) hemorrhages; (2) diffuse and limited contusion of the brain; (3) laceration of the brain; (4) secondary inflammations of the brain and arachnoid membranes (almost invariably pyogenic). Here again the information given the reader is so minute and extensive that only this general statement of its excellence can be given. The same remark is true of the chapters upon diagnosis, prognosis and treatment. Regarding the treatment, we cannot refrain from quoting the author concerning the operative treatment of the various lesions previously discussed :

If in the general class of intracranial injuries operation is to be but infrequently done, the question of operation will be often raised and decision as to the course to be pursued will then entail grave responsibility, since error in judgment may deprive the patient of a chance for life by increasing the danger of an already critical condition. Action or inaction at the wrong moment has invited disaster on either hand ; but instances of too early or unwarranted operative interference by inexperienced surgeons outnumber those in which the ultraconservatism of their elders has led to a perhaps fatal neglect.

Later, regarding the operative treatment when encephalic lesions are independent of accessible cranial fracture, the author makes the following wise remarks :

Operation is not to be done as a so-called last resource, and because the patient is likely or sure to die without it, as he is with it, unless some blind chance interposes where reason affords no room for hope. Intracranial exploration will be defensible, as it is made with or without sufficient cause, and not as it may conform to an opinion deducible from a wide generalisation of results that it is a good or a bad procedure. Unless general rules can be made absolute, the obligation to determine in each instance the treatment to be pursued in accordance with the indications which it presents remains unimpaired, and the contention that more lives are lost by operative interference which is unnecessary than by its neglect when it is required has no relation to the exigencies of particular cases.

The general management of these cases is limited to the fulfilment of the indications directly afforded by symptoms, and include stimulation, cold to the head, mechanical restraint, control of nervous irritation and maintenance of strength of the individual.

Part II. deals with pistol-shot wounds, first in their medico-legal and second in their surgical relations.

The author, believing that a very great number of observations systematically made is essential to the formulation of rules which govern the infliction and reception of this type of gunshot wounds, made a large number of experiments upon the cadaver in which the caliber of the ball, its angle of incidence, the distance traversed by it, the length of the weapon, the character of the explosive, the density and thickness of the cranium and the special region involved were studied. The calibers selected were 0.22, 0.32, 0.38 and 0.44. The cartridges were of a single manufacture and the distances the same for each caliber, varying from absolute contact to 100 feet. Additional observations were made with pistols and cartridges of other makers, and also with smokeless in place of the black powder. The trials were made upon entire subjects recently dead, where the conditions present conformed as nearly as possible to those in the living.

The effects of pistol-shot wounds upon the head especially studied were lesions of the tegumentary coverings and of the cranium.

Intracranial lesions possessed for the author less certain value because of possible modifications due to post-mortem changes.

The specific effects noted included for the external soft parts the characteristics of the external wound of entrance, the burning of the skin or hair, smoke stains of the skin and the deposit of unburned grains of powder upon the surface or in the substance of the skin or subjacent wounded tissues ; and for the cranium, the peculiarities of the osseous wounds of entrance and of exit and the resulting fractures of the vault or base. To these were added an examination of the brain track for powder traces or bony fragments. The results obtained are concisely given and the various lesions admirably illustrated from photographs taken at the time of the experiments.

We cannot take the space to record even the generalisations made by the author from these experiments, but it may be safely affirmed that they are so complete and accurate that they will be used and quoted by medico-legal experts in the future.

Of pistol-shot wounds in their surgical relations, the author speaks in the same clear and concise way. The intracranial lesions characteristic of these wounds are contusion, laceration and hemorrhage.

Of symptoms the most common is unconsciousness, which is always present in the immediately fatal cases, and is unusually profound. For purposes of diagnosis the probe is the main reliance. Girdner's telephone probe is commended in suitable cases. The finger, where it can be used, fulfils the office of a probe admirably. Of the X-rays, the author's opinion is that as yet they are not of especial value, but have possibilities. The use of the sharp needle is advocated where for any reason the probe or finger is not sufficient. Summing up concerning the diagnostic means within our reach Dr. Phelps says :

> These several methods of search, guided by the observation of existing localising symptoms or of indications of injury to the opposite cranial wall, are the only means available for the discovery of the bullet. Their successful use requires not only manual skill, but quickness of perception and sagacity of interpretation in the study of the often obscure attendant conditions.

The treatment is divided into general and local, the former being in the main the same as applicable in similar cases produced by other forms of violence. The local treatment includes thorough cleansing, removal of fragments, incision of dura, when indicated, or when doubt as to its necessity exists, extraction of the bullet whenever possible, for the reason that it is usually septic, and even when this is not so Dr. Phelps regards it as likely to produce dural or cerebral irritation in the future. The treatment is concluded by a discussion of the methods and limitations and the details of the various procedures recommended. The book concludes with the condensed histories of 300 intracranial traumatisms selected from 500 original cases.

The type and general dress of the work and the admirable illustrations are in accord with the scientific excellence of the work. It should be in the hands of every surgeon and medico-legal expert. J. P.

